# Health system to response to economic sanctions: global evidence and lesson learned from Iran

**DOI:** 10.1186/s12992-022-00901-w

**Published:** 2022-12-29

**Authors:** Haniye Sadat Sajadi, Reza Majdzadeh

**Affiliations:** 1 Knowledge Utilization Research Center, University Research and Development Center, Tehran University of Medical Sciences, Tehran, Iran; 2 School of Health and Social Care, University of Essex, Colchester, UK

**Keywords:** Global health, Health Policy, Universal Health Care, Economic Recession, Iran

## Abstract

**Background:**

Sanctions have direct and indirect impacts on people’s lives. Therefore, the health systems of countries targeted by sanctions must respond effectively. This study proposes a set of mitigating measures and response strategies to improve the health systems of countries under sanctions.

**Methods:**

This three-stage study was conducted in Iran within the 2020–2021 period, in which a rapid review of evidence was carried out to identify the measures implemented or proposed to make the health system resilient in confronting sanctions. A qualitative approach was then adopted to determine how the health system could be improved to response to sanctions from the perspectives of 10 key experts. Semi-structured interviews and document analysis were conducted for data collection. Finally, a two-round Delphi technique was employed to help eleven experts reach a consensus on a set of mitigating measures, which were then prioritized.

**Results:**

In this research, 62 proposed or implemented mitigating measures were extracted from 13 eligible studies to improve the health system performance in confronting sanctions. Moreover, 18 measures were identified in interviews for a better health system response to sanctions. They were then classified as five categories: sustained financing, good governance, integrated and updated health information systems, qualified workforce, and efficient and equitable service delivery. In the first Delphi round, 28 mitigating measures were discovered. Nine measures were identified as more effective and feasible in both short and long runs. They were introduced as below: conducting proactive inventory control, developing the nationally essential list of medicines, providing additional clarification that oil revenues can be freely used for medicines procurement, defining tailored health service packages for vulnerable populations, establishing and enhancing an efficient surveillance system, reducing prices of imported medicines, developing dual policies of equity and priority for vulnerable groups, institutionalizing fair and effective resource allocations, and providing clinical guidelines.

**Conclusions:**

According to the findings, the most critical areas for the resilience of a health system in confronting sanctions include strengthening particular components of governance, improving efficiency, and caring for vulnerable populations. The experts collectively emphasized investment in domestic capacities, public participation, and health diplomacy. Despite the proposed measures, it is unclear how effective these are and, especially whether they can significantly affect the harsh impacts of sanctions on health. Moreover, intensive and long-term sanctions have significant irreversible outcomes that cannot be reversed easily or quickly.

**Supplementary Information:**

The online version contains supplementary material available at 10.1186/s12992-022-00901-w.

## Background

Iran has implemented remarkable initiatives to improve its health system and provide all citizens with accessible healthcare services. These services include expanding the primary healthcare network, integrating medical education programs, training and recruting family physicians, and developing health transformation plans. These efforts have led to significant changes in the public health and improvements in Iran’s health system performance (Table 1). Nevertheless, various threats affect this system. Global threats (e.g., emerging and re-emerging diseases and impacts of climate change), the Middle East issues (i.e., conflicts and wars), nationwide natural disasters, and international sanctions are the main factors that threat Iran’s health system [1].

**Table 1:** Selected health system indicators of Iran (https://data.worldbank.org)

Indicator	1980	2020
Life expectancy at birth (year)	54	77
Maternal mortality ratio (per 100,000 live births)		16^b^
Neonatal mortality rate (per 1,000 live births)	40	8
Infant mortality rate (per 1,000 live births)	85	11
Under-five mortality rate (per 1,000 live births)	108	13
Immunization, measles (% of children ages 12–23 months)	39	99
Immunization, DTP (% of children ages 12–23 months)	32	99
Current health expenditure (% of GDP)		6.7^a^
Out-of-pocket expenditure (% of current health expenditure)		40^a^
Hospital bed density (per 1,000 people)	1.5	1.6^b^
Physician density (per 1,000 people)	0.3	1.6^c^
Nurses and midwives (per 1,000 people)		2.1^c^

Latest data available, if not 2020: ^a^2019; ^b^ 2017; ^c^2018

In this regard, political tensions and sanctions are the ongoing potential threats impacting Iran’s health system, especially because they have been intensified for several decades. Various entities have imposed different sanctions against Iran since 1979 [2]. Apart from the negative outcomes of sanctions, Iran’s health system has severely been affected [3]. Although the previous round of sanctions imposed by the United Nations (UN) Security Council against Iran did not concern essential medicines, equipment, and commodities, it caused a 65% reduction (16,000/1 to 26,500/1) in Iran's Rial to the US Dollar exchange rate during a year, 30% inflation (https://www.cbi.ir), and subsequently more than a one-third decrease in the public purchase power to cover health expenditures [4]. As a result, the under-utilization of health services emerged in the early 2010s in Iran. Allegedly, the imports of finished products, pharmaceutical raw materials [5], and medical devices [6, 7] were disrupted due to bans on the connection between Iranian banks and SWIFT (i.e., Society for Worldwide Interbank Financial Telecommunications) and on their trades with the global banking system. These bans increased prices of medicines by half [8] and caused the shortage of nearly 73 medicines. Moreover, 48% of these medicines are classified as essential by the World Health Organization (WHO).

It is also estimated that six million patients faced limited access to necessary treatments [9]. According to a report on cases of thalassemia and haemophilia, there were approximately 60% and 90% shortages of medicines for patients [10]. Another study on diabetes, asthma, cancer, and multiple sclerosis indicated that there were significant shortages in 13 out of 26 medicines due to sanctions [11]. Evidently, the poor and disadvantaged people were more vulnerable and affected severely in all cases of shortages. A recent review demonstrated that sanctions had deprived Iranians of the human right to health [12].

Evidently, sanctions have adverse effects on the public health and cause significant financial hardships in the accessibility of healthcare services. These outcomes often face the individuals that belong to marginalized and vulnerable groups. Sanctions also degrade health systems, especially with regard to the availability of healthcare services. They can also negatively affect health research and education. Although the evidence is scarce for the quantification of potential effects left by sanctions on different dimensions of the public health, sanctions have negative outcomes in this regard [13, 14].

Despite Iran’s long history of sanctions, there is inadequate evidence regarding how the health system responds effectively and which mitigating measures are necessary for managing the effects of sanctions [15]. Given the endurance of sanctions against Iran and their direct and indirect effects on people’s lives [3, 16], the health system must respond efficiently to this inconvenience. In fact, a health system should be able to adapt to such pressures or be resilient to the harsh effects of sanctions. The resiliency of a health system refers to the capacity to absorb changes and retain essential functions [17]. The key questions pertain to how well the health system has continued to operate in the face of sanctions and how well vulnerable groups have been protected. Hence, this study proposes a set of mitigating measures and response strategies to improve Iran’s health system performance in the face of sanctions. Furthermore, the proposed measures and strategies are relevant to various countries where economic sovereignty is threatened.

## Methods

**Fig. 1:**
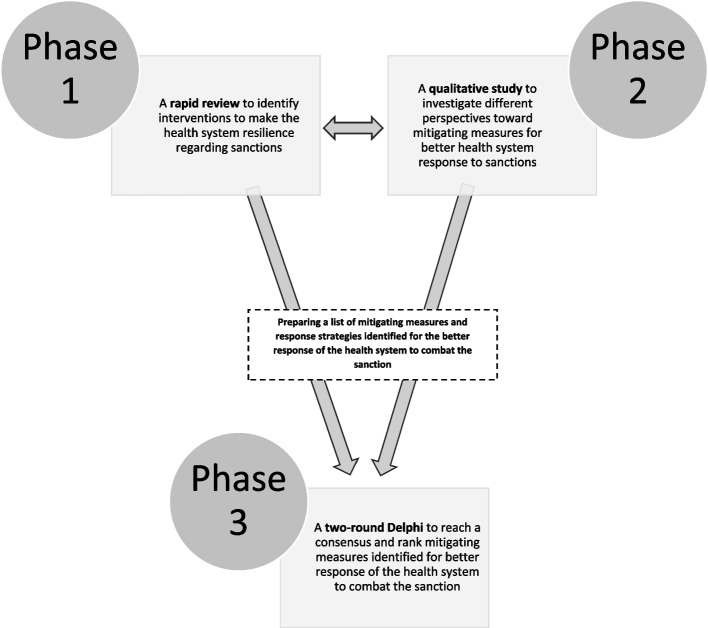
Overview of three stages of the study

This study employed the mixed-methods (qualitative–quantitative) research design within the 2020–2021 period in the following stages (Fig. 1):

### Stage one

A rapid evidence review was conducted to identify the measures or interventions implemented or proposed to make health systems resilient in response to sanctions. Rapid reviews are among the emerging methods of efficiently synthesizing research evidence in health policies and other settings where a broad overview of research evidence is required quickly [18]. For this purpose, the PRISMA reporting guidelines were adopted in this study. The search strategy was developed in consultation with a librarian and, for the rapid review, was limited to two databases due to time and resource limitations. One author (HSS) searched two English databases (i.e., PubMed and Scopus) to find relevant studies. An additional search attempt in Google Scholar was made with exact keywords in order not to miss any relevant documents. The search strategy was restricted to the English language, and the peer-reviewed papers were published from the inception of these databases. The initial search attempt was made in 2020 and was then updated in 2021. Two broad concepts were selected to ensure that the search strategy addressed the research question: sanction (e.g., embargo, economic sanction, and financial shock) and health (e.g., medical and medicine). For the full search strategy, please refer to Additional file 1: Appendix 1.

Studies were eligible for inclusion if they reported on primary or secondary research and provided sufficient data regarding the strategies implemented or proposed to improve the health system performance in response to sanctions. Other types of publications (e.g., notes, editorial, commentary, letter, conference proceedings, etc.), studies related to economic/financial crises or shocks due to reasons other than sanction, and studies for which full texts were unavailable were excluded.

Citations were downloaded to EndNote© X9 (Thomson Reuters, New York, USA). Two authors (i.e., HSS and RM) evaluated titles and abstracts and excluded the irrelevant ones. Disagreements were then resolved by consensus. The authors obtained and reviewed full texts of the remaining citations, excluding the ones that did not meet the inclusion criteria. They hand-searched the reference lists of the remaining papers for additional relevant studies.

Data extraction was performed by authors using a standardized form, which included the title, first author’s name, publication date, setting, research design, the method used, details of participants, and main findings related to the implemented or proposed measures to make the health system resilient in response to sanctions. A narrative synthesis was employed to analyze the findings of the included studies. Furthermore, the WHO’s Health System Framework [19] was then modified to create tables and interpret findings.

### Stage two

The rapid review was supplemented with a qualitative study to triangulate findings and validate our understanding. It was conducted in Iran through a phenomenological approach, aiming to analyze the perspectives of key experts about mitigating measures and strategies to improve the health system in response to sanctions. Semi-structured face-to-face interviews and document analysis were conducted for data collection.

Purposeful sampling with a maximum diversity method was adopted to select participants, who were the experts familiar with health systems, health policymakers, and managers, particularly during the Iran–Iraq war, financial recessions, and other similar situations. The interviews were conducted through the guidelines developed based on the review results. The participants were asked to express their ideas on what was needed for the effective health system response to sanctions and share their experiences about the mitigating measures and strategies of Iranian authorities to respond to sanctions. These participants were informed prior to the meeting objectives via phone calls or emails. Their conversations were audio-recorded with their consent. Notes were also taken during the discussion. The audio files were then transcribed verbatim. Summaries of the interviews/meetings were emailed to participants for revision and completion if necessary. Moreover, relevant documents such as newspapers, rules, regulations, and formal reports were reviewed.

The manifest content analysis was used by one author (HSS) for data analysis, in which we described what the experts said, stayed very close to the text, used the words themselves, and described the visible and obvious in the text [20]. Discussions resolved disagreements, and coding was conducted through inductive and deductive approaches. We also considered the quality criteria such as credibility, dependability, reflexivity, transferability, and confirmability. We then engaged with all participants to build the necessary levels of trust. We also adopted the data triangulation strategy. External audits were then employed to ensure dependability. Working on data collection and analyses, researchers considered the process of critical self-reflection about preferences and preconceptions to guarantee reflexivity. The transferability of the study was ensured by selecting the appropriate experts to participate in interviews. Confirmability was achieved by obtaining the opinions of some participants (i.e., member check).

#### Stage three

A two-round Delphi process was adopted to reach a consensus and rank a set of mitigating measures and response strategies identified to help the health system respond better to sanctions. We selected relevant experts from different stakeholder groups with experience or expertise in the health system, policy, and sector reform. They included policymakers and academic members with publications on sanctions. The expert panel members were identified through purposive sampling strategies. As a result, 24 potential experts were identified and double-checked by the research team. Finally, based on the accessibility and affordability of sufficient time for participation, 15 eligible experts were extracted. A brief illustration of the study was distributed to them, and they were invited to the Delphi process via emails. Apart from two, all experts accepted to participate in the study: one declined to participate, and the other could not be contacted. The willing ones were provided additional material about the study and the research method.

The Delphi process was performed in 2021 with intervals of three weeks between the first and second rounds. Based on the findings of previous stages, some mitigating measures was prepared. In the first Delphi round, a cover letter was emailed to all experts, who were asked to suggest additional measures that they found important and included in prioritization. The experts individually reflected their opinions and re-sent their comments within a due date (15 days). A reminder was then emailed a few days before the deadline to maximize the response rate. We reviewed their comments and finalized the mitigating measures.

Based on the first round of Delphi, a questionnaire was developed. In fact, it was a pilot tested with five health specialists and academicians to examine coherence and face validity before administration. The final questionnaire included 28 mitigating measures and two ranking criteria (i.e., effectiveness and feasibility in both the short and long runs). The experts individually completed the ranking for reflecting their opinions on a 5-point Likert scale (from “1” for “least priority” to “5” for “highest priority”) and re-sent the questionnaire within a due date (15 days). A reminder was emailed a few days before the deadline to maximize the response rate. The characteristics of the participants were also asked on the first page of the questionnaire. The questionnaire was emailed to all experts to invite them to rank each measure based on the prioritization criteria (i.e., feasibility and effectiveness).

In order to analyze the second Delphi round, the average score of each criterion for overall measures was determined (note: the average and median scores were nearly identical). These estimates were made for the short and long terms separately. Depending on whether the average score of each measure is higher or lower than the overall average in terms of two criteria (i.e., feasibility and effectiveness), the measures were then classified as four categories: I) effective and feasible, II) effective but not feasible, III) feasible but not effective, and IV) neither effective nor feasible. These categories were used for the final interpretation of measures.

## Results

### Stage one

**Fig. 2:**
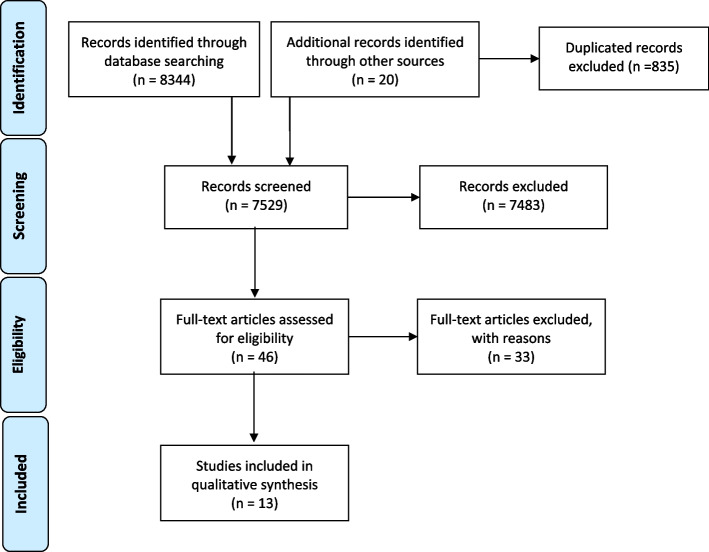
PRISMA flow chart of search, inclusion, and exclusion screening, and accepted studies of the review on measures or interventions implemented or proposed to make the health system resilient regarding sanctions

We identified 7437 studies, out of which 13 met the inclusion criteria (refer to Additional file 1: Appendix 2). The PRISMA diagram outlines the screening process (Fig. 2). Most studies were conducted in Iran and published after 2010. They included eight reviews and five original studies. The review output was a list of 62 proposed or implemented mitigating measures to improve the health system performance in response to sanctions. Table 2 presents an overview of the measures.

**Table 2:** Summary of mitigating measures to improve the performance of the health system in terms of sanctions identified via review

The targeted function of the health system	Mitigating measures	Source	Type of measure
Financing arrangement	1. Mobilizing latent resources in education and health	[21]	Implemented
	2. Providing additional clarification that Iranian oil revenues can be freely used for medicines procurement without reservations	[9]	Proposed
	3. Supporting local production	[22, 23]	Implemented
	4. Price reduction of imported medicines	[22]	Implemented
	5. Optimizing the domestic market	[15]	﻿Proposed
	6. Centralized and strategic purchasing	[23]	﻿Proposed
	7. Rationalization of the prices of medicines	[23]	﻿Proposed
	8. Strengthening the insurance system	[23]	﻿Proposed
Governance arrangement	9. Establishing uniform criteria and definitions for exemptions as well as operational criteria for sanctions committees to facilitate improved effectiveness of exemptions	[24]	﻿Proposed
	10. Developing dual policies of equity and priority for vulnerable groups	[25]	Implemented
	11. Mobilizing public participation to compensate for reduced access to capital goods	[21]	Implemented
	12. The use of public systems to motivate behavioral change, with a focus on the needs of women and children	[21]	Implemented
	13. Refocusing health policy toward maximizing scarce resources	[21]	Implemented
	14. Professional organizations, especially those concerned with children's health, must advocate for children in countries experiencing economic sanctions	[26]	﻿Proposed
	15. Advocating for global health diplomacy to ensure that ongoing multilateral negotiations do not neglect public health and humanitarian need	[9, 27]	﻿Proposed
	16. Establishing regulatory export harmonization	[9]	﻿Proposed
	17. Amending the OFAC EAR99 classification system to make it easier for US companies to export medicines	[9]	﻿Proposed
	18. Exempting vaccine products from stringent export controls	[9]	﻿Proposed
	19. Allocating a protected SWIFT line specifically for humanitarian medicines trade	[9]	﻿Proposed
	20. Exempting medicine and medical commodities from “snap back” provisions;	[9]	Proposed
	21. Providing a national policy with measures to prevent the suffering of people from the adverse effects of sanctions	[12]	﻿Proposed
	22. Preventing third parties; black market dealers, pharmacies, and health facilities that provided unsafe medicines, as well as smugglers who sent scarce medicine to neighbouring countries	[12, 23]	﻿Proposed
	23. Refraining from imposing embargos and other measures that restrict the supply of medicine and medical equipment	[12]	﻿Proposed
	24. Developing policies and laws to alleviate the negative impacts of their agreements on the human rights of the population in the target country	[12]	﻿Proposed
	25. Designing some international intermediate organizations and certain companies and financial institutions to facilitate the implementation of exemptions	[12]	﻿Proposed
	26. Permitting immediate release of medicines from customs by minimum financial documents	[22]	Implemented
	27. Determining a memorandum of understanding (MOU) between the IFDA Iran’s Food and Drug Administration) and the Central Bank	[22]	Implemented
	28. Determining a memorandum of understanding (MOU) between the IFDA and health insurance organizations for the extra financial protection of special, incurable, and chronic patients with high pharmaceutical expenditures and for allocating the additional budget to over-compensate unaffordable pharmaceutical products based on the equity-based criteria	[22]	Implemented
	29. Developing the national essential medicines list	[22]	﻿Proposed
	30. Using use all available political and legal means, such as health diplomacy, to establish humanitarian channels to enhance global convention and remove possible barriers as the sanctions and reduce their adverse consequences for antimicrobial resistance control	[28]	﻿Proposed
	31. Creating efficient food assistance programs by the government and the international community, funding food banks with the assistance of charities and non-governmental organizations, and participating individuals in nutritional education programs and learning how to plan a cheap and balanced diet	[29]	﻿Proposed
	32. Boosting the morale, knowledge, skills, and innovation of managers can potentially increase resilience	[15]	﻿Proposed
	33. Creating mutual trust among different organizations	[15]	﻿Proposed
	34. Revisions in Iran’s health management	[23]	﻿Proposed
	35. Delegation and privatization	[23]	Proposed
	36. Strengthening of inter-sectoral cooperation	[23]	Proposed
	37. More attention to mass media	[23]	﻿Proposed
	38. Interactions with neighbouring countries	[23]	Proposed
	39. Management and development of health tourism	[23]	﻿Proposed
Information and evidence	40. Improving means of monitoring the impact of sanctions on civilian populations in targeted countries, particularly concerning water purity, food availability, and infectious disease control	[24]	﻿Proposed
	41. Advocating prospective studies to generate the data needed to provide better information and monitoring capacity than presently exists	[24]	﻿Proposed
	42. Strengthening health monitoring systems	[21, 23]	Implemented
	43. Advocating for the integration of Health Impact Assessments (HIAs) that identify the health consequences of sanctions	[9]	﻿Proposed
	44. Monitoring human rights situations and utilizing the maximum resources available to eliminate suffering with low-cost programs, international assistance, and cooperation	[12]	﻿Proposed
	45. Assess the effects of the policies and international agreements on the health of people in the target country	[12]	﻿Proposed
	46. Observing the situation of human rights and implementing humanitarian and human rights laws	[12]	﻿Proposed
	47. Employing cost-effectiveness evidence for pricing and reimbursement	[22]	﻿Proposed
	48. Developing an integrated information system for monitoring the market	[22]	﻿Proposed
	49. Determining the exact magnitude of the impact	[30]	﻿Proposed
	50. Electronic health record	[23]	﻿Proposed
	51. Electronic prescription	[23]	﻿Proposed
Service delivery arrangement	52. Rationing, universal access to primary health care, a highly educated population, and preferential access to scarce goods for women and children	[25]	Implemented
	53. Emphasizing preventative over curative medicine	[21]	Implemented
	54. Protecting vulnerable groups of the population, such as children and the poor	[12]	﻿Proposed
	55. Facilitating the delivery of necessary items for life and health, such as medicine, food, and medical equipment	[12]	Proposed
	56. Proactive inventory control	[22]	Implemented
	57. Providing clinical guidelines for rational prescribing	[22, 23]	﻿Proposed
	58. Proper implementation of the referral system	[23]	﻿Proposed
	59. Medication tracking	[23]	﻿Proposed
	60. Informing the medical community	[23]	Proposed
	61. Use of alternative medicines and methods	[23]	Proposed
	62. Consumer–patient collaboration	[23]	﻿Proposed

### Stage two

Ten respondents completed the interview. The details of participants can be found in Additional file 1: Appendix 3. According to many participants, Iran has always faced sanctions, except for a limited time.

*“Iran is a country that is actually under sanctions, and many of these sanctions are related to the foreign policy, and the first place that is affected by the sanctions is usually the health sector, and the first place that benefits from the lifting of the sanctions is the health sector, too.” P1.*Although efforts to delegitimize sanctions should not be overlooked, the health system must prepare for sanctions to operate better and minimize the damage caused by sanctions.

*“At least a series of measures should be taken to delegitimize the system of sanctions in the health sector and show the effects of sanctions on the health sector as well as the violation of human rights and basic rights of Iranian citizens.” P1.*

They highlighted several measures required to make the health system more resilient to the effects of sanctions. In fact, 380 meaningful codes were extracted, and 18 subcategories were identified (mitigating measures proposed by participants to improve the health system performance in response to sanctions). They were then classified as five main categories (i.e., health system functions) (Table 3).

**Table 3:** Mitigating measures to make a health system resilient to sanctions identified via interviews and gray literature

Category (targeted function of the health system)	Subcategory (mitigating measures)
Sustained financing	1. Using sustained health financial resources 2. Fair and effective resource allocations 3. Systematic costing of medicines and medical devices
Good governance	4. Preparedness and planning for sanction 5. Collateral collaborations for procurement of goods 6. Optimizing/shrinking organizational structure 7. Investing in domestic production 8 Strong leadership and management 9. Having constant collaboration and active social networks 10. Empowering the community and increasing their participation 11. Strengthening the health diplomacy
Integrated and updated health information system	12. Constant monitoring and evaluation 13. Enhancing surveillance system 14. Strengthening evidence-informed policymaking
Qualified workforce	15. Provision of adequate skilled health workforce 16. Motivating health workforce
Efficient and equitable service delivery	17. Giving priority to public health intervention 18. Defining tailored health service packages for vulnerable populations

### Sustained financing

#### Using sustained health financial resources

Nearly all participants viewed the availability of resources as a key enabler in making a health system resilient. They said that the health system could overcome disruption when available resources were used strategically. Financial resources are necessary to mobilize other essential resources during sanctions and crises. The sustained protection of healthcare funding was identified as a key ingredient to its resilience. Given the economic status of Iran which mainly depends on oil, it was strongly recommended that diverse and stable financial resources, e.g., taxation or further efficiency, should be employed to minimize the risk of an underfunded response.

“You know that national income depends on the sales of oil in Iran. Whenever there is a disturbance in the sales of oil, almost all sectors suffer. The health sector is no exception. We know this, and its effects are also obvious. Now we have to ensure that, as far as possible, the financial resources of the health sector have minimal dependence on oil revenues. We should think about finding more sustainable resources for the health system.” P2.

“Our health system should be able to withstand sanctions and make some changes to be less vulnerable during sanctions. For example, do you remember that after the JCPOA, Iran’s income improved a bit, and as a result, the financial resources of the health sector increased? The same health transformation plan was implemented at the same time. It would be better if the health system tried to use these funds to become efficient so that it would not be under pressure if the embargo happened again. For instance, more should be invested in the primary health than hospitals.” P7.

#### Institutionalizing fair and effective resource allocations

Participants stated that the sanctions strongly affected how government resources were allocated to health and how health resources were allocated to various programs. In the absence of priorities and resource allocations, the shares of health expenditures usually reduce, and many health plans fail to be implemented, both of which are considered constant threats. The participants pointed out some of the efforts made in Iran to use a scientific and fair approach for prioritization and allocation of resources in the health sector. However, they believed that these efforts did not lead to the institutionalization of a clear and accurate method for prioritization. Thus, an approach to institutionalized and effective setting priorities and financial resource allocation should be developed to make a health system more resilient.

“The embargo there has led to a decrease in our incomes, and economic austerity has been formed after that. These austerity attempts will affect the allocation of resources to our government departments. When the allocation of resources declines, the policies of the Ministry of Health will be revised. How? This revision should be based on the principles of prioritization. Still, because there is no systematic prioritization, priorities are formed based on preferences, which often causes a waste of resources. We need a system that determines how to spend this little money more effectively and efficiently.” P10.

#### Systematic costing of medicines and medical devices

According to the participants, the most direct effect of sanctions was left mainly on the prices of medicines, medical devices, and equipment. The increased prices of these items have limited access or resulted in the emergence the black market, leading to the poor quality of these items, all of which pose a serious health risk. Many participants highlighted the vulnerability of medicines and medical equipment during the embargo period and the importance of selling medicines and medical products at stable prices during sanctions.

“We now know the pricing of some medicines, which means it is clear how much this medicine should cost. The same is true for some medical equipment and devices, but this price list is not for all items and is sometimes not updated. If we could have an updated list of prices for drugs and medical equipment available to all Iranian people, in times of crisis like this embargo, traders could not sell drugs to people at any price.” P5.

### Good Governance

#### Preparedness and planning for sanction

Some participants stated that sanctions and their outcomes had not properly been analyzed in Iran’s national health planning and policies and that goals and strategies were not organized accordingly. In addition, although a resistant economy is an appropriate solution to counter the effects of sanctions, its implementation has not seriously been pursued. The participants emphasized that health planners should consider the scenario of continuing sanctions, and any targeting should be done in accordance with sanctions and their effects. The scenario exercises were reviewed, and pseudo-sanction situations were mentioned as a strategy to make preparations for crises or disasters.

“Sanctions will happen now, this round, or the next round. You must have a plan to confront sanctions, and until then, there will never be any discussions of sanctions. We did not look at any outlooks that sanctions might happen while we are forecasting. We did, but we did not consider the embargo. First, we did not look at it from this point of view. We never included these facts related to the embargo in our work, and this is a big problem that the country has.” P7.

#### Collateral collaborations for procurement of goods

Some participants said that during sanctions and the restrictions imposed on financial transactions and transfers, one of the most important factors that could be effective in maintaining the health system was the use of alternative ways of conducting exchanges and procurement resources. Ensuring that the health system has multiple alternative courses of action can lead to resilience. Collateral pathways refer to the availability of alternative routes to achieve the desired goal and enhance resilience by providing alternative courses of action. When a system experiences disruption or challenges on one pathway, an alternative pathway is utilized to achieve the same goal.

“There is a special bandage that the … company produces only in …, and they no longer sell these to Iran due to sanctions, and we saw what we did and the follow-up we did. We pressured the … ambassador, that is, the … government through … lawyers and in a way forced the … factory to sell, it was not their way, then they said we would not sell to the Ministry of Health, then we said there was no problem, sell it to … Iran, we will give it to the Ministry of Health. Then, because these issues of financial transfer and other things were difficult to solve, … made a donation and got a budget, and with the help of the ... government and the first party, we bought the first party.” P4.

#### Optimizing/shrinking organizational structure

Another governance practice affecting the resilience of a health system is an appropriate organizational structure to cope with sanctions. Participants believed that it was essential to establish a flexible and agile structure for deciding how to respond to sanctions and monitor the proper implementation of policies.

“After all, one of the emerging issues is the structure. Do the structures change because of this? Some things happened. For example, a deputy was removed. … Or, for example, assume that the Food and Drug Administration changed its organizational arrangement, and the issue of combating pharmaceutical problems and managing corruption was somehow brought up in the Ministry of Health.” P10.

#### Investing in domestic production

Another governance issue affecting the resilience of the health system is the formulation of simple rules and regulations away from bureaucracy to support domestic production. Some participants described the experience of eliminating cumbersome regulations in support of the domestic production of medicines and equipment, arguing that a transparent and law-abiding approach to supporting domestic production could reduce the dependence of the health system on foreign countries. As a result, they suffered less from sanctions.

“It can be said that one of the advantages of the embargo is that domestic production has been revived. We produce a lot of vaccines and medicines inside Iran. The more we can strengthen our internal capacities and potential to be producers, the less we will lose during sanctions. Of course, domestic production must be of high quality.” P2.

#### Strong leadership and management

The importance of managerial and leadership practices to the resilience of the health system is a recurrent concept emphasized by many participants. In a resilient health system, management and leadership are characterized by inclusive decision-making. Participants stated that managers and leaders should ensure that relevant stakeholders were included and contributed to decision-making. This could nurture the resilience of the system by building trust and empowering, motivating, and creating commitment among staff and other stakeholders.

“We have a lot of experience with this. For example, some ministers and deputies turned their words into actions during the war. Regarding sanctions, it is not possible for the minister not to speak or not to be heard. Look at the COVID-19 pandemic. Whenever they made a decision, they asked the Minister of Health because health is important. Even for sanctions, the words of those who know the bad outcomes for people's health cannot be heard. Health managers need to be charismatic and decisive.” P2.

#### Having constant collaboration and active social networks

Many participants stressed the importance of a government’s comprehensive efforts and people’s resistance to sanctions. They mentioned how well the health system established and leveraged its networks to determine its resilience to everyday challenges and acute shocks. Social networks offer useful avenues for the increased mobilization and transfer of knowledge and dissemination of innovations, thereby boosting the overall resilience of systems. Collaboration among organizations in a networked environment can also expand potential resources, the ability to learn, and the capacity to respond.

“If you remember the war, these jihadi groups and popular networks played an important role in helping soldiers and supporting the country in many places. Since they had a social base, they could coordinate resources well. The health system should also consider how it can create and improve these networks and communities in the community groups. It is a kind of social capital that can give morale in difficult situations and can do many things instead of the government.” P6.

#### Empowering the community and increasing their participation

According to the notes by participants, community empowerment is the strongest way to make the health system resilient. Community participation is a kind of investment in health, and it helps achieve huge capital.

“Undoubtedly, if the society is empowered, the health system can work more effectively, especially in crises. Do you know how much nongovernmental organizations can improve our drug shortages? The truth is that I think if we can work on this community participation, we can get through crises like embargo much better.” P10.

#### Strengthening health diplomacy

Some participants mentioned the benefits and advantages of health diplomacy, believing that it was a fundamental approach to resolving disputes between nations. Therefore, as a part of the plan to respond to sanctions and make the health system more resilient, public policymakers should consider their population and global health issues in their relationships with other countries. For success in health diplomacy, it is advisable to provide solid evidence to release the effects of sanctions on the human rights of target countries. The evidence can delegitimize sanctions in the international community and be used as the basis for international policies to improve global health.

“This all goes back to the field of diplomacy and health diplomacy or the same thing you are doing now. How do you challenge sanctions? This goes back to health diplomacy because the health field is an area that can easily interact. It means that even in the most difficult situations when countries are at war, it is easier to enter from the health sector, interact, and enter into the cooperation process. This field is the field of diplomacy and health.” P1.

### Integrated and updated health information system

#### Constant monitoring and evaluation

According to the participants, the missing link of appropriate response to sanctions is the lack of a monitoring and evaluation system to analyze the effects of sanctions on health. Referring to data and studies currently available in Iran, they said that, unfortunately, due to the lack of a codified system, the data could not be used to judge the type and severity of impacts left by sanctions. Sometimes, the data required to perceive the impacts of sanctions does not exist. Another issue is the lack of scientific methodology to analyze the effects of sanctions on health. These all result in the wake of proactively monitoring what is happening in the health system. Equity was highlighted as an aspect that is often lost during sanctions. One participant stated that new strategies made hastily in need of a quick fix would often miss equity aspects. Hence, it is highly important to establish a monitoring and evaluation system in which different aspects of equity are routinely observed.

“The point was that we never came to see these indicators, how our access is to medicine, equipment, and procedures during the embargo, and the effect of the embargo on the public health was not there to see what their conditions would be. This makes us not have a complete judgment on this whole issue.” P7.

“Indicators of malnutrition in children, thinness, short stature, and even overweightness and obesity in children and adults were among of the important indicators that could be affected by sanctions. I did not see very documented statistics. One of the claims made by the Ministry of Health was that we kept it constant, and these indicators had not changed.” P8.

#### Enhancing surveillance system

The resilience of the health system is widely identified as dependent on how health data and information are managed and used. All participants believed that data and information including routine health or administrative data, valid and accurate research evidence, and survey data are the key to how timely and adequately the health system adapted to challenges caused by sanctions. Moreover, many participants highlighted the need for adequate information and epidemiological surveillance systems that monitor and report the status of the system and provide real-time early warning of impending health threats.

“When they create a surveillance system and want to control an epidemic, first of all, you have to see if the prevalence is high or if the economic burden is high, that is. I mean we have criteria for saying which should be the surveillance system. Now in Iran, many information systems are excessive. To be more resilient, we need a surveillance system as needed. For the same women's urine, it is possible to study whether this has changed during the embargo or their treatment and access to treatment have changed.” P7.

#### Strengthening evidence-informed policymaking

According to the participants, there are many obstacles in helping policymakers make evidence-informed decisions or sue for the illegitimacy of sanctions. The most important problems included not paying attention to monitoring the effects of sanctions on health and the health system; lack of registration and reporting systems; scattered, inaccurate or contradictory statistics, especially in the field of food status and access to health services; and low quality and the incompleteness of some of the evidence produced in Iran about the effects of sanctions. These obstacles must be solved to make the health system resilient. Adaptive and new strategies should be developed based on previous experiences, and lessons learned should efficiently be implemented.

“This is an area that I think you should document in this case, especially in international authorities, to put pressure on this, on this field, and in fact, the argument regarding the international legitimacy of the US sanctions system as a whole. A distinction was made between banking sanctions and sanctions in the health sector.” P1.

### Qualified Health Workforce

#### Provision of adequate skilled health workforce

Participants recognized the important role that health workforces would play in the resilience of the health system. They pointed out that having an adequate number of health workforces and the requisite skills were critical contributors to resilience.

“It can be good. Sanctions affect the dollar exchange rate, which will harm the outflow of human resources, which means that they are more in demand. See, if we are looking to strengthen the health system, especially we, who are always at risk of sanctions, human resource policies should be changed a bit. We should learn what other societies are doing.” P9.

#### Motivating health workforce

It is essential to guarantee that the health staff is adequately motivated and fully committed to the predetermined goals. A way of ensuring that health workforces are motivated and committed is to prioritize their well-being. It was achieved by creating a positive social environment where the staff was free to express emotions and share information, providing adequate resources to match their work demand, actively listening, monitoring, and addressing changing staff stresses, and flexibility around staff needs.

“There are some factors that are more fundamental and not easily seen, attention to the incentive system, attention to the payment system, attention to the various human resource systems in the health system. These important issues are practically neglected. if they can be used for a strong health system, they can also create motivation, that which we feel in your presence, belonging to patriotism.” P9.

### Efficient and equitable service delivery

#### Giving priority to public health intervention

Nearly all participants emphasized that establishing the primary health network was very helpful and supportive in providing essential health services for the population in recent decades. They strongly suggested focusing on preventive efforts such as public health interventions during stable times as one strategy for a health system to be well-prepared for sanctions. However, most sanctions and shocks were difficult to predict and prevent.

“The most serious damage caused by sanctions is for drugs and medical supplies mainly used for treatment. The conditions of the patients have worsened with every sanction. It is very simple, for the health system not to be harmed by this issue, for it should focus on prevention. As you said, prevention had priority over treatment at the beginning of the revolution.” P6.

#### Defining tailored health services package for vulnerable populations

Some participants stated that during sanctions, not only is it important to provide routine health services for the entire population, but it is also essential to ensure additional health services for vulnerable groups including women, children, low socioeconomic population, and refugees. However, maintaining everyday services should be put high on the agenda by a resilient health system. They assumed that defining and developing tailored health service packages might help ensure safe and premium care with minimum financial hardships.

“Experience has always shown that those with incurable diseases and those whose voices are less heard are more harmed during crises. For instance, if patients know some people in high places, their voices will be heard; otherwise, nobody listens to them. Now, suppose the Ministry of Health has the claim of trying to achieve equity in health. In that case, it should sit down and see where the vulnerable groups are in terms of health in society and what minimum services should be provided for them in crises to monitor them regularly.” P7.

#### Stage three

**Fig. 3:**
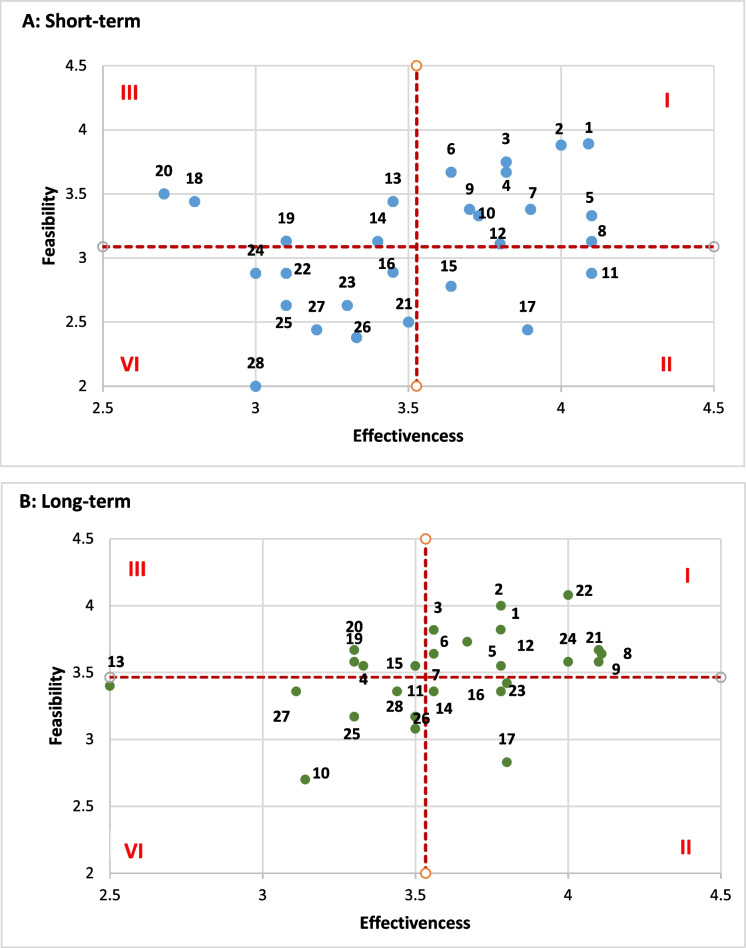
Ranking mitigating measures for health system resilience against imposed sanction (based on expert opinions in Delphi rounds). The red dotted line shows the overall average score for each axis. Based on these areas are defined as: I. Effective and feasible. II. Effective but not feasible. III. Feasible but not effective. IV. Neither effective nor feasible

Thirteen experts were invited to join the panel. Finally, eleven responded. Additional file 1: Appendix 3 presents further details regarding the expert panel used in this study. In the first Delphi round, 28 mitigating measures were found once the suggestions and comments of experts were assessed. In the second Delphi round, all these measures were prioritized with regard to their effectiveness and feasibility. Table 4 presents the measures and their relationships with the functions of the health system.Fig. 3 A–B reports the ranking results, and Table 5 demonstrates the classification of resultant interventions based on the average scores of feasibility and effectiveness criteria in accordance with the time horizon of effects. For the sake of simplicity, any scores above the overall average score were considered effective or feasible, and the scores below average were considered ineffective or non-feasible.

**Table 4:** The mitigating measures for the resilience of Iran’s health system

Code	Mitigating measures	Health System Function towards resilience	Source of data	Delphi
1	Proactive inventory control	Efficient and equitable service delivery	Review	Accepted
2	Developing the list of nationally essential medicines	Efficient and equitable service delivery	Review	Accepted
3	Providing additional clarification that Iranian oil revenues can be freely used for medicine procurement without reservations	Sustained financing	Review	Accepted
4	Using the capacity of some international intermediate organizations and certain companies and financial institutions to facilitate purchasing medical items	Good governance	Review	Accepted
5	Defining tailored health service packages for vulnerable populations	Efficient and equitable service delivery	Interview and gray literature	Accepted
6	Establishing and improving a strong surveillance system	Integrated and updated health information system	Review & Interview and gray literature	Accepted
7	Price reduction of imported medicines through public resources	Sustained financing	Review	Accepted
8	Developing dual policies of equity and priority for vulnerable groups	Good governance	Review	Accepted
9	Institutionalizing fair and effective resource allocations	Sustained financing	Interview and gray literature	Accepted
10	Considering collateral pathways for procurement of required medical items	Good governance	Review & Interview and gray literature	Accepted
11	Extra financial protection for special, incurable, and chronic patients and for allocation of the additional budget to over-compensate unaffordable pharmaceutical products	Sustained financing	Review	Accepted
12	Providing clinical guidelines for rational prescribing	Efficient and equitable service delivery	Review	Accepted
13	Establishing an appropriate organizational structure to deal with the sanction	Good governance	Review & Interview and gray literature	Accepted
14	Having constant collaboration and active social networks at both national and global levels	Good governance	Review & Interview and gray literature	Accepted
15	Prioritizing health among public policies	Sustained financing	Review	Accepted
16	Strengthening evidence-informed policymaking	Good governance	Review & Interview and gray literature	Accepted
17	Preventing third parties, black market dealers, pharmacies, and health facilities that provide unsafe medicines as well as smugglers	Good governance		Accepted
18	Facilitating immediate release of medicines from the customs with minimum financial documents	Service delivery	Review	Accepted
19	Adapting exportation laws based on domestic needs	Good governance	Review	Accepted
20	Conducting Health Impact Assessments (HIAs) that identify the effects of sanctions on healthcare	Good governance	Review	Accepted
21	Founding for health via sustained sources	Sustained financing	Interview and gray literature	Accepted
22	Investing in domestic production	Sustained financing	Review & Interview and gray literature	Accepted
23	Empowering the community and increasing their participation	Good governance	Review & Interview and gray literature	Accepted
24	Strengthening the global health diplomacy	Good governance	Review & Interview and gray literature	Accepted
25	Establishing support mechanisms to prevent and control the social harms of the economic outcomes of sanctions (e.g., the protection of working children)	Efficient and equitable service delivery	Review	Accepted
26	Improving the system for fair and effective allocation of resources between health plans and relevant executive bodies in health	Sustained financing	Review & Interview and gray literature	Accepted
27	Institutionalizing economic evaluation of medicines, medical devices and equipment, and health services	Sustained financing	Review & Interview and gray literature	Accepted
28	Optimizing the use of human resources (by improving competencies and making appropriate use of job descriptions, e.g., avoiding specialization in basic services)	Qualified workforces	Interview and gray literature	Accepted
29	Preparedness and planning for sanction	Good governance	Interview and gray literature	Rejected
30	Strong leadership and management	Good governance	Review & Interview and gray literature	Rejected
31	Optimizing the domestic market	Sustained financing	Review	Rejected
32	Strengthening the insurance system	Sustained financing	Review	Rejected
33	Strengthening of inter-sectoral cooperation	Good governance	Review	Rejected
34	Paying more attention to mass media	Good governance	Review	Rejected
35	Management and development of health tourism	Good governance	Review	Rejected
36	Electronic health record	Integrated and updated health information system	Review	Rejected
37	Electronic prescription	Integrated and updated health information system	Review	Rejected
38	Proper implementation of the referral system	Efficient and equitable service delivery	Review	Rejected
39	Medication tracking	Efficient and equitable service delivery	Review	Rejected
40	Consumer–patient collaboration	Efficient and equitable service delivery	Review	Rejected

**Table 5:** Classification of interventions based on the average scores of feasibility and effectiveness criteria in accordance with the time horizon of effects

Category	Mitigating measures	
	**Short term**	**Long term**
I: Effective and feasible	12	11
II: Effective but not feasible	4	3
III: Feasible but not effective	4	5
IV: Neither effective nor feasible	8	9

Comparing these two timeframes resulted in valuable findings, among which it is noteworthy to pay attention to three groups of mitigating measures.

- Group 1: Effective and feasible measures in both short-term and long-term periods. Most of these measures focus on good governance and strengthening and improving supervision.
- Group 2: Measures that are more effective in the short run than the long run.
- Group 3: Measures that are more effective and feasible in the long run than the short run (e.g., funding for health through sustained sources, investing in domestic production, and strengthening global health diplomacy).

Apart from these measures, we identified some other measures that should be considered in the international community and agencies under sanctions (e.g., strengthening global health diplomacy, assigning some international intermediate organizations and specific companies and financial institutions to facilitate the implementation of exemptions, excluding vital medicine and medical supplies from sanctioned items, allocating a protected specific banking channel for humanitarian medicines trade, establishing specific cut-off thresholds for unintended consequences on civilians, establishing an international order for the protection of people before the imposition of sanctions, and monitoring and evaluation the effects of sanctions).

## Discussion

In this study, some mitigating measures and response strategies were identified to improve the performance of Iran’s health system in response to sanctions. These measures can benefit countries under sanctions. However, also agencies such as the UN overlook human rights to reduce the health burden of sanctions.

The review findings indicated the insufficient evidence on measures improving the health system to cope with the effects of sanctions and to harness the outcomes. They are mostly derived from the synthesis of studies on available reports and statistics, and there was no robust evidence on their effectiveness and implementation considerations. Therefore, it is not easy to judge the effectiveness and feasibility of these measures. Further investigations are required to extend the existing knowledge about the effectiveness of these measures and to find context-based implementation considerations.

Research findings also revealed a range of measures proposed or implemented to make a health system resilient to sanctions. These measures focus on strengthening governance arrangements, better information and evidence, sustained financing, qualified health workforce, and efficient and equitable service delivery. Most of these measures are similar to those recommended for health system rebuilding or preparedness. For instance, Palagyi et al. explored and introduced the key elements of health systems in low- and middle- income countries during the outbreak as (i) surveillance, (ii) infrastructure and medical supplies, (iii) workforce, (iv) communication mechanisms, (v) governance, and (vi) trust [31]. Hanefeld et al. also reported that three health systems functions would help the health system to adapt and respond to shocks: (i) health information systems (having the information and the knowledge to decide on what needs to be done), (ii) funding/financing mechanisms (investing or mobilizing resources to fund a response), and (iii) health workforce (who should plan and implement the system) [32]. A recent brief policy published by Thomas et al. [33] reported the same findings to introduce strategies for enhancing the resilience of health systems. Therefore, different shocks experienced by the health systems brought the idea that health systems needed to be not only stronger but also more resilient [32]. We need to reinforce the interconnectedness of the traditional health system building blocks to respond better.

The findings, both at the review and qualitative phases, indicated that most measures to make a resilient health system in sanctions were related to strengthening governance. It highlights the critical role of the system software (i.e., governance arrangements) in enabling health systems to cope with the critical situation. It is similar to the ones reported by previous studies. Douedari and Howard considered health system governance elements (i.e., strategic vision, participation, transparency, responsiveness, equity, effectiveness, accountability, and information) to play central roles in rebuilding the Syrian health system in conflicts [34]. Hanefeld et al. also introduced governance as a fundamental function affecting all other system dimensions and predominant values shaping the response and how it would be experienced at individual and community levels [32]. We believe that governance is not a standalone function. In fact, it is the mortar that binds all other functions together. Thus, it is not easy to make the health system more resilient. It is also not an apolitical technical exercise. In other words, it is a rather intensely complex task that needs complex interventions.

As mentioned earlier, there was scant evidence regarding the effectiveness of measures. Therefore, we obtained the opinions of experts on the effectiveness and feasibility of measures identified in studies and the qualitative phase. Considering the similarities of effectiveness and feasibility of measures in the short run and the long run, we found that more effective and applicable measures in the short run and the long run were those the ones that increased the efficiency of health resources and the correct use of resources, leading to making a resilient health system against sanctions. Several studies have concluded that the main issue in all health systems is inefficiency and the waste of health resources [35, 36]. Thus, it is essential to ensure efficiency improvement to make a resilient health system cope with catastrophic events. In addition, the other effective and feasible measures to cope with a tough situation included the measures related to defining well-defined policies and ensuring the continuity of services to vulnerable people such as children and the poor, and monitoring the impact of sanctions on their condition in targeted countries. Regarding the emphasis on equity and the prism of leaving no one behind in health [37, 38], taking these measures to improve equity is a critical step in making the health system more sustained and responsible.

Evidently, although shortcut ways to deal with the sanction that rely on the capacity of international organizations or sideways to bypass the sanction might be suitable in the short run, they fail contribute sufficiently to the long-term resilience of a health system. While taking these measures are inevitable in the short term, it is essential to plan for more effective measures. Regarding the research findings, measures to increase national capacities are a part of effective measures in the long run. It has also been recommended to rely on national capacities since the PHC declaration [39]. One of the four PHC principles is the use of appropriative technology. It refers to any technology that makes the most economical use of a country’s natural resources and its relative proportion of capital, labor, and skills and contributes to national and social goals. Thus, it is recommended to invest in boosting national capacities and reducing the health system’s needs abroad.

Additionally, measures to empower the community and increase participation are more effective in the long run. Investment in social participation starts with building government capacities to create, manage, and sustain participation mechanisms. More public participation enables the health system to be more secure [40]. Finally, we recognized that it would be vital to promote the view of health as an international issue and remove it from a nationalistic perspective. Developing a novel innovative health diplomacy approach must incorporate a multidisciplinary political framework that includes human rights [2]. It is recommended that governments monitor the human rights situation in the targeted countries and utilize the maximum resources available to eliminate shortages with low-cost programs, international assistance, and cooperation.

## Conclusions

Although sanctions have a long history, there is a scarcity of empirical studies on the approaches required to mitigate the health effects of sanctions and make the health systems more resilient to cope sanctions. Countries might have valuable experiences but are less reported as peer-reviewed publications. According to the results of this study, the most critical area for the resilience of a health system in confronting sanctions is to strengthen particular components of governance. The experts collectively believed that improving efficiency and caring for vulnerable populations were more effective and feasible. They mentioned that while prompt measures were required to cope with catastrophic circumstances, effective measures were more vital in the long run. These measures include investing in national capacities rather than waiting for foreign aid, empowering people, and strengthening health diplomacy. Despite the novelty of the proposed measures, it is unclear how effective they are and, in principle, whether they can significantly affect the harsh effects of sanctions on health. Moreover, intensive and long-term sanctions leave significant irreversible outcomes, which cannot be removed easily or in the short run.

## Supplementary Information

**Additional file 1: Appendix 1.** Search strategy. **Appendix 2.** Overview of studies included in the rapid review. **Appendix 3.** Respondent characteristics in interviews and Delphi rounds.

## Data Availability

The datasets used and/or analyzed during the current study are available from the corresponding author upon reasonable request.

## Abbreviations

SWIFT: Society for Worldwide Interbank Financial Telecommunications
UN: United Nations
WHO: World Health Organization
