# Supplementary material for: Health system to response to economic sanctions: global evidence and lesson learned from Iran
Source: Global Health. 2022 Dec 29;18:107. doi: 10.1186/s12992-022-00901-w (PMC9797877; doi:10.1186/s12992-022-00901-w)
Supplement: Supplementary file 1 — Additional file 1: Appendix 1. Search strategy. Appendix 2. Overview of studies included in the rapid review. Appendix 3. Respondent characteristics in interviews and Delphi rounds. [file 12992_2022_901_MOESM1_ESM.docx]

# **Additional file 1**

# **Appendix 1: Search strategy**

**PubMed**

("sanction*"[Text Word] OR "embargo*"[Text Word] OR "economic shock*"[Text Word] OR "financial shock*"[Text Word] OR "financial hardship*"[Text Word] OR "financial crisis"[Text Word] OR "economic crisis"[Text Word]) AND ("health"[Text Word] OR "medic*"[Text Word])

**Scopus**

( TITLE ( sanction*  OR  embargo*  OR  "economic shock*"  OR  "financial shock*"  OR  "financial hardship*"  OR  "financial crisis"  OR  "economic crisis" )  AND  TITLE-ABS-KEY ( health  OR  "medic*" ) )

# **Appendix 2: Overview of studies included in the rapid review**

| **Title** | **First author** | **Year** | **Type of the study** | **Setting** |
| --- | --- | --- | --- | --- |
| The Health Impact of Economic Sanctions | Garfield R | 1995 | Review | Several nations |
| The Impact of the Economic Crisis and the US Embargo on Health in Cuba | Garfield R | 1997 | Review | Cuba |
| The Public Health Impact of Sanctions: Contrasting Responses of Iraq and Cuba | Garfield R | 2000 | Review | Cuba and Iraq |
| Addressing the impact of economic sanctions on Iranian drug shortages in the joint comprehensive plan of action: promoting access to medicines and health diplomacy | Setayesh S | 2016 | Review | Iran |
| Assessment of the Effects of Economic Sanctions on Iranians' Right to Health by Using Human Rights Impact Assessment Tool: A Systematic Review | Kokabisaghi F | 2018 | Review | Iran |
| Policies to improve access to pharmaceutical products in shortage: the experience of Iran food and drug administration | Yousefi N | 2019 | Original | Iran |
| Control of antimicrobial resistance in Iran: the role of international factors | Mehtarpour M | 2020 | Original | Iran |
| Sanctions on Iran and their impact on child health | Madani-Lavassani Y | 2020 | Review | Iran |
| The Effects of the Re-imposition of US Sanctions on Food Security in Iran | Hejazi J | 2020 | Original | Iran |
| Economic sanctions and child health | Choonare | 2013 | Review | Iran |
| How Sanctions Have Impacted Iranian Healthcare Sector: A Brief Review | Akbarialiabad H | 2021 | Review | Iran |
| Resilience to economic sanctions; case study: Hospital equipment cluster of Tehran (HECT) | Taslimi MS | 2021 | Original | Iran |
| Strategies to improve pharmaceutical supply chain resilience under politico-economic sanctions: the case of Iran | Bastani P | 2021 | Original | Iran |

# **Appendix 3: Respondent characteristics in interviews and Delphi rounds**

| Participants Characteristics | Face-to-face semi-structured interview  (N= 10) | Two-round Delphi participants  (N=11) |
| --- | --- | --- |
| Gender |  |  |
| Female | 3 | 3 |
| Male | 7 | 8 |
| Age |  |  |
| 40-49 years | 1 | 2 |
| 50-60 years | 6 | 7 |
| > 60 years | 3 | 2 |
| Work experience |  |  |
| 10-19 years | 3 | 2 |
| 20-30 years | 5 | 7 |
| > 30 years | 2 | 2 |
| Literacy status |  |  |
| Master of sciences/art | 3 | - |
| Medical Doctor | 2 | 2 |
| Doctor of Philosophy | 5 | 9 |
| Expertise |  |  |
| Foreign policy | 1 | - |
| International relations in health | 2 | 2 |
| Health system | 5 | 6 |
| Faculty members and researchers | 1 | 3 |
